# *Delftia tsuruhatensis,* an Emergent Opportunistic Healthcare-Associated Pathogen

**DOI:** 10.3201/eid2403.160939

**Published:** 2018-03

**Authors:** Alexandre Ranc, Grégory Dubourg, Pierre Edouard Fournier, Didier Raoult, Florence Fenollar

**Affiliations:** MEPHI, UMR, IRD, Aix-Marseille Université, Institut Hospitalo-Universitaire Méditerranée Infection, Marseille, France (A. Ranc, G. Dubourg, D. Raoult);; VITROME, Institut Hospitalo-Universitaire Méditerranée Infection, Marseille (P.E. Fournier, F. Fenollar)

**Keywords:** *Delftia tsuruhatensis*, emergence, pneumonia, bacteremia, healthcare-associated, pathogen, bacteria, France

## Abstract

*Delftia tsuruhatensis,* which was first isolated in environmental samples, was rarely associated with human infections. We report on pneumonia caused by *D. tsuruhatensis* in an infant who underwent cardiac surgery. Retrospective analyses detected 9 other isolates from 8 patients. *D. tsuruhatensis* is an emergent pathogen, at least for immunocompromised patients.

*Delftia tsuruhatensis,* a member of the *Comamonadaceae* family, was first isolated from sludge in Japan in 2003 (*1*). Mainly studied for environmental purposes (*2**,**3*), *D. tsuruhatensis* has rarely been identified in humans (*4**,**5*)*.* We present a case report of a respiratory infection caused by *D. tsuruhatensis* in a premature infant.

A female infant, born premature at 36 weeks’ gestation, had a cardiac congenital pathology for which resection of the ductus arteriosus and pacemaker placement were performed at 4 months of age. During the immediate follow-up period, she developed acute renal failure, which was treated by peritoneal dialysis. Her undernutrition status required enteral and parenteral nutrition. Laboratory tests showed slight leukocytosis, with elevated neutrophils at 9.2 G/L (reference range 1.4–8.5 G/L), monocytosis at 2.1 G/L (reference 0.2–2.0 G/L), and an elevated C-reactive protein at 15.1 mg/L (reference 0–5 mg/L). Two days after surgery, the infant developed pneumonia associated with ventilator-associated hypoxia, which prompted bronchial aspiration sampling that was sent to the clinical microbiology laboratory for analysis, which was performed as previously described (*6*)*.* Colonies grew after 24 hours’ incubation on both Polyvitex and Columbia media (bioMérieux, Craponne, France) in pure culture at 10^7^ CFU/mL. We correctly identified the isolate using matrix-assisted laser desorption/ionization time-of-flight (MALDI-TOF) mass spectrometry (Microflex, Bruker, Leipzig, Germany), as previously described (*7*). We maintain a custom MALDI-TOF mass spectrometry database that is updated regularly and enabled identification of the colony as *D. tsuruhatensis*, with an identification score of 2.082. We confirmed identification of the strain using 16S rDNA amplification coupled to sequencing, as previously reported (*8*). We obtained an amplicon of 1,296 bp and identified it as *D. tsuruhatensis* with a similarity of 99.70% with GenBank sequence no. KC572558. Because the strain was negative for d-mannitol assimilation as highlighted using API NE (bioMérieux), we excluded possible misidentification with *D. lacustris.*

We tested for antimicrobial drug susceptibility according to EUCAST 2017 recommendations (*9*) using the Etest gradient method. We categorized the strain as resistant to amoxicillin (MIC >256 mg/L) and amoxicillin/clavulanate (MIC >256 mg/L) but susceptible to ceftriaxone (MIC 0.5 mg/L), ertapenem (MIC 0.5 mg/L), imipenem (MIC 0.5 mg/L), and ofloxacin (MIC 0.047 mg/L). We administered ceftazidime to the patient for 10 days. Further collected samples were negative on culture. 

One month later, the infant became febrile (temperature 39°C); a chest radiograph revealed pneumonia, and testing showed a still-elevated C-reactive protein (15 mg/L). A new bronchial aspiration was obtained and inoculated, as described previously. Twenty-four hours after incubation, we observed 2 isolates, each growing 10^5^ CFU/mL, and analyzed them by MALDI-TOF mass spectrometry. One isolate was identified as *D. acidovorans* (score 2.207). Faced with the discrepancy with the previous results, we performed 16S rRNA PCR coupled with sequencing, as previously reported, enabling the identification of *D. tsuruhatensis* with a sequence identity of 99.70% to GenBank sequence no. KC572558. We identified the other isolate as *Neisseria macacae*, with a score of 2.033.

We initiated therapy with imipenem, vancomycin, and amikacin before we received the microbiology results, after which we readjusted the regimen, this time administering only tobramycin aerosol. The patient’s health gradually deteriorated; she developed bradycardia and refractory hypoxia. She died at 6 months of age, 12 days after the last isolation of *D. tsuruhatensis.*

We report isolation of *D. tsuruhatensis* in respiratory samples from a 6-month-old infant, born at 36 weeks’ gestation. Recurrent isolations of the microorganism from the same patient, including 1 time in pure culture, exclude potential contamination. In addition, clinical signs, such as pneumonia with ventilator-associated hypoxia, support infection rather than colonization. However, the patient had recurrent pneumonia, despite a successful first therapy with ceftazidime.

We also looked at the number of strains of *D. tsuruhatensis* isolated in our university hospitals in Marseille, France, during 2008–2015 and in the literature (Table). The microorganism has been isolated 13 times from 11 patients, including the case we describe here, mainly from blood cultures (5/11 cases) and respiratory specimens (5/11), but also from 1 urine sample. Overall, the underlying conditions were observed for 10 cases, including 2 transplant recipients. No information was available for 1 patient. Considering the presence of a vascular catheter, hospital stay longer than 48 hours, or both, all reported infections were healthcare associated. In addition, of the 6 patients in whom the bacterium had been isolated from blood cultures, all 6 had an intravascular device. These data are consistent with the 2 cases of bacteremia involving *D. tsuruhatensis* already reported in the literature for which intravascular device–related and underlying conditions were found (*4**,**5*)*.* Bacterial identification systematically failed when using phenotypic methods. Since its implementation in routine laboratory tests, MALDI-TOF mass spectrometry has correctly identified *D. tsuruhatensis* in 4 of 8 tested isolates. For the 4 other isolates, *D. tsuruhatensis* was misidentified as *D. acidovorans* in 3 cases. Accurate identification was definitively performed using 16S rDNA sequencing.

**Table:** Characteristics of 11 patients with *Delftia tsuruhatensis* infection, 2 from the literature and 9 from university hospitals in Marseille, France*

Year, patient age, y/sex	Underlying conditions	Intravascular device	Specimen (description)	Clinical features; drug regimen	ID method; ID (score)	Amplification of 16 rDNA (similarity)	Ref
2010, 53/F	Metastatic breast cancer	Yes	Blood culture	Port-related bacteremia with fever; ceftriaxone	Phenotypic methods; *Comamonas testosteroni*	*D. tsuruhatensis* (99%)	(*4*)
2012, 53/F	Severe pulmonary hypertension	Yes	Blood culture	Catheter-related bacteremia with chills; oral ciprofloxacin	Phenotypic methods; *D. acidovorans*	*D. tsuruhatensis* (100%)	(*5*)
2008, 77/M	Liver cancer, colic adenocarcinoma	Yes	Bronchial aspirate (10^5^ CFU/mL, pure)	Considered by physicians as colonization	Phenotypic methods; *D. acidovorans*	*D. tsuruhatensis* (100%)	Marseille hospitals (this study)
2009, 70/F	Unknown	Unknown	Bronchial aspirate (10^5^ CFU/mL, pure)	Not available	MALDI-TOF MS; *D. acidovorans* (1.968)	*D. tsuruhatensis* (99.9%)	Marseille hospitals (this study)
2010, 59/F	Alcoholism, chronic end-stage renal failure	Yes	Blood culture	Catheter-related bloodstream infection; piperacillin, tazobactam, gentamycin	Not available	*D. tsuruhatensis* (99.9%)	Marseille hospitals (this study)
2010, 6/M	Cystic fibrosis	No	Sputum (10^3^ CFU/mL, not pure)	Not available	MALDI-TOF MS; *Arthrobacter castelli*	*D. tsuruhatensis* (99.1%)	Marseille hospitals (this study)
2013, 42/M	Homeless, chronic renal failure, alcoholic hepatitis	Yes	Urine (10^6^ CFU/mL, pure)	Not available	MALDI-TOF MS; *D. tsuruhatensis* (2.19)	*D. tsuruhatensis* (99.8%)	Marseille hospitals (this study)
2014, 13/F	Liver transplant	Yes	Blood cultures (N = 2)	Post-transplant fever; piperacillin, tazobactam	Not available	*D. tsuruhatensis* (99.9%)	Marseille hospitals (this study)
2015, 47/M	Kidney transplant	Yes	Blood culture	Fever	MALDI-TOF MS; *D. tsuruhatensis *(2.38)	Not performed	Marseille hospitals (this study)
2015, 82/M	Hemodialysis, vascular dementia	Yes	Blood culture 1	Catheter-related bloodstream infection; ceftazidime	MALDI-TOF MS; *D. acidovorans* (2.02)	*D. tsuruhatensis* (100%)	Marseille hospitals (this study)
			Blood culture 2		MALDI-TOF MS; *D. tsuruhatensis* (2.21)	*D. tsuruhatensis* (100%)	
2015, <1/F	Preterm birth	Yes	Respiratory sample 1 (10^7^ CFU/mL)	Ventilator-associated pneumonia; ceftazidime, second-line treatment with imipenem and amikacin	MALDI-TOF MS; *D. tsuruhatensis* (2.08)	*D. tsuruhatensis* (99.9%)	Marseille hospitals (this study)
			Respiratory sample 2 (10^5^ CFU/mL)		MALDI-TOF MS; *D. acidovorans* (2.21)	*D. tsuruhatensis* (100%)	

*Ref, reference; ID, identification; MALDI-TOF MS, matrix-assisted laser desorption/ionization time-of-flight mass spectrometry.

In conclusion, *D. tsuruhatensis* is an opportunistic emergent healthcare-associated pathogen that can be easily misidentified. Clinicians should consider this bacterium particularly in immunocompromised patients and those with intravascular devices.
